# Fine-Scale Patterns of Population Stratification Confound Rare Variant Association Tests

**DOI:** 10.1371/journal.pone.0065834

**Published:** 2013-07-04

**Authors:** Timothy D. O’Connor, Adam Kiezun, Michael Bamshad, Stephen S. Rich, Joshua D. Smith, Emily Turner, NHLBIGO Exome Sequencing Project, ESP Population Genetics, Statistical Analysis Working Group, Suzanne M. Leal, Joshua M. Akey

**Affiliations:** 1 Department of Genome Sciences, University of Washington, Seattle, Washington, United States of America; 2 Program in Medical and Population Genetics, Broad Institute, Cambridge, Massachusetts, United States of America; 3 Division of Genetics, Brigham and Women’s Hospital, Harvard Medical School, Boston, Massachusetts, United States of America; 4 Department of Pediatrics, University of Washington, Seattle, Washington, United States of America; 5 Center for Public Health Genomics, University of Virginia School of Medicine, Charlottesville, Virginia, United States of America; 6 Department of Molecular and Human Genetics, Baylor College of Medicine, Houston, Texas, United States of America; The University of Chicago, United States of America

## Abstract

Advances in next-generation sequencing technology have enabled systematic exploration of the contribution of rare variation to Mendelian and complex diseases. Although it is well known that population stratification can generate spurious associations with common alleles, its impact on rare variant association methods remains poorly understood. Here, we performed exhaustive coalescent simulations with demographic parameters calibrated from exome sequence data to evaluate the performance of nine rare variant association methods in the presence of fine-scale population structure. We find that all methods have an inflated spurious association rate for parameter values that are consistent with levels of differentiation typical of European populations. For example, at a nominal significance level of 5%, some test statistics have a spurious association rate as high as 40%. Finally, we empirically assess the impact of population stratification in a large data set of 4,298 European American exomes. Our results have important implications for the design, analysis, and interpretation of rare variant genome-wide association studies.

## Introduction

Population structure can be a strong confounding factor in association studies [1]–[4], and accounting for it can be important, even in cases where seemingly homogeneous ethnic populations are sampled. For example, low yet detectable levels of population structure have been reported in samples from Icelandic [5], British [6], and French Canadian populations [7] as well as in European Americans [8], [9]. The effects of population structure on association tests have largely been explored in the context of common genetic variation [6], [10]. However, as common variants have been unable to account for a significant proportion of complex disease heritability [11], [12], there is increasing interest in systematically evaluating the contribution of rare variants to disease.

To this end, a large number of rare variant association test statistics have been developed (reviewed in Bansal et al. [13] and Asimit and Zeggini [14]) and used to identify a growing catalog of rare alleles that may influence disease risk [13], [14]. One of the main statistical approaches used to date has been collapsing of rare variants in order to increase statistical power over single variant tests [13], [15], [16]. However, collapsing of rare variants also has the potential to increase the power to detect associations due to population stratification. Furthermore, previous studies have shown that large sample sizes are needed to obtain sufficient power to robustly associate rare variants with complex traits [13], [17], thus increasing the likelihood of sampling individuals from populations with unrecognized structure. We will refer to the elevation in or inflation of significance rates as the spurious association rate (SAR) throughout the rest of the paper to emphasize the point that population stratification causes genuine associations between genotypes at a locus and a phenotype, but such associations are due to genetic substructure rather than alleles causally related to the trait.

Two recent studies have explored how rare variant association methods perform in the presence of population stratification. Tintle et al. [18] used exon pilot data of the 1000 genomes project [19] and found that, as expected, the SAR is inflated when associations are performed in samples drawn from geographically diverse populations. Specifically, their analysis was performed on individuals of Asian, European, and African ancestry pooled together. They found that taking covariates from a principal component analysis (PCA) was generally sufficient to reduce the SAR. A second study by Mathieson and McVean [20] developed a biogeographic model where phenotypic outliers are sampled from one geographic locale. Similar to Tintle et al. [18], Mathieson and McVean [20] primarily focused on the effects of global population structure, which leads to high SARs. Interestingly, PCA was unable to correct for population stratification in the biogeographic model, illustrating that particular types of structure are more difficult to control for in association studies. Mathieson and McVean [20] also extended their models to more modest levels of population structure, and found qualitatively similar results.

Although these two studies have provided insights into the behavior of rare variant association studies in the presence of population structure, several important questions remain. In particular, the quantitative impact of fine-scale population structure is not well defined. Indeed, as large sample sizes are necessary to detect associations with rare variants [13], [17], [21], [22], fine-scale population structure is likely to be present in many datasets. Moreover, there have been no systematic analyses of how sensitive different rare variant association methods are to population structure. To address these issues, we comprehensively evaluated the robustness of nine rare variant association methods to modest levels of population structure. Further, we investigated how the power of the methods changes when in the presence of population structure and empirically assessed the SAR in a large exome dataset [17].

## Materials and Methods

### Rare Variant Association Methods

We evaluated nine rare variant association methods: the collapsed χ^2^ test, the collapsed Fisher’s Exact Test (FET), the Weighted Sum Statistic (WSS) [23], Variable Threshold (VT) [24], RareCover [25], and four methods implemented under a logistic regression framework. The logistic regression methods include a collapsed variant test [15] where variants with a MAF<1% are collapsed into a single class (i.e. *logit Y = α+β_α_*×*I*, where *I* is encoded as 1 for any variant with a MAF≤1% or 0 for no such variant), a StepUp test [26], a StepDown test (different from StepUp only in optimization strategy), and the Combined Multivariate and Collapsing (CMC) method that analyzes common and rare variants jointly, but as separate covariates [15]. These methods were implemented in a Java program CCRARE, which is available at http://akeylab.gs.washington.edu/downloads.html, and uses the library Math Commons 2.1 (http://commons.apache.org/math/index.html). We define a variant as rare if it has a MAF≤1%, and except for CMC and VT, are the only variants analyzed. For a detailed description of each method see Table S1 in File S1.

For all analyses, statistical significance was determined empirically using permutations. We performed 1,000 permutations for each test statistic unless specified otherwise, which is sufficient to evaluate a α = 0.05 significance threshold. For computational efficiency, we used a rejection procedure, which stops permuting once more than α×1,000 statistics are greater than the test statistic. The p-values are thus not estimated in the full 1,000 permutations, but the approximation is useful for testing significance at a particular threshold [23], [25]. All p-values were calculated as (k0+1)/(k +1) where k0 are the number of permutations with a more significant test statistic than the original test and k are the current number of permutations calculated. The test was stopped when k0≥ α×1,000. We used a α of 5% for computational efficiency given the large parameter space we are exploring.

### Methodological Theory for Confounding

To simulate a confounding effect due to population structure, we adapt a previously described approach [2], [27]–[29]. To calculate the proportion of cases and controls that are sampled from each subpopulation, we allowed differences in disease risk between subpopulations where the probability an individual has a disease, *P(d = c|i)*, is conditional on the *i* ^th^ subpopulation. Furthermore, we denote the probability that an individual is drawn from the *i* ^th^ subpopulation as *P(i)*. Intuitively, *P(i)* can be interpreted as the proportion of the sample that is selected from subpopulation *i*, thus, 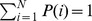. Using Bayes theorem, we can calculate the probability of drawing an individual from the *i* ^th^ subpopulation given either case (*c*) or control (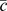) status as:
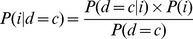
(1)where
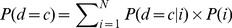
(2)
[29] and N is the number of subpopulations. To obtain the analogous probabilities for controls, c is substituted with 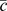 in the above equations. For any given subpopulation, 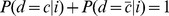.

For each simulated scenario, we randomly paired haplotypes within each subpopulation to produce diploid individuals. Unless otherwise noted, we randomly sampled without replacement 1,000 cases and 1,000 controls from the subpopulations based on their disease probability:
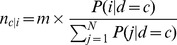
(3)where *n_c|i_* is the number of cases out of *m* (1,000 unless otherwise noted) that come from the *i^th^* subpopulation.

### Calibrating a Demographic Model

**Figure 1:**
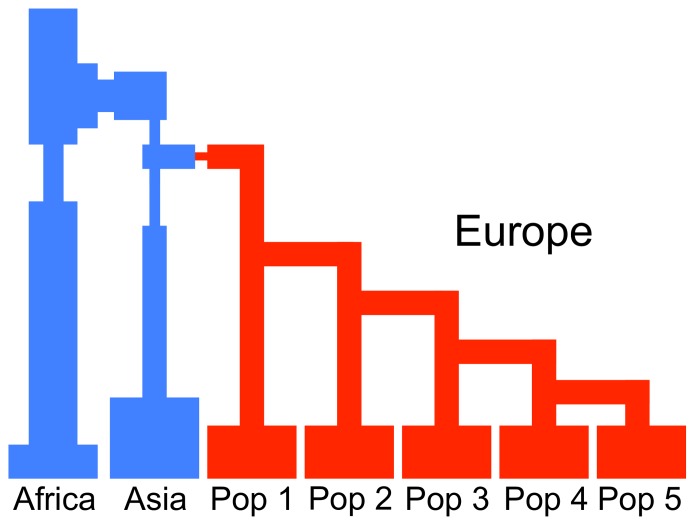
Schematic of demographic model used in the simulations. Parameter values were inferred by calibrating to patterns of variation in exome data from 316 European Americans.

We used the strategy of Schaffner et al. [30] to calibrate a demographic model with European population structure (see Figure 1 and Figure S1A in File S1) that approximates empirical patterns of DNA sequence variation. We chose a five-subpopulation model as this provides flexibility in studying how prevalence differences, levels of differentiation, and ascertainment strategies influence rare variant association methods. The simulation parameters were initially calibrated to 316 European American exomes sequenced as part of the NHLBI Exome Sequencing Project (ESP; http://snp.gs.washington.edu/EVS/) [17]. We calculated the root mean square error (RMSE), an estimate of goodness of fit, between observed and simulated data for the following statistics: linkage disequilibrium (r^2^) (with bins of nucleotide distance spaced by 100 kb sections), Tajima’s D, nucleotide diversity (π), and the site frequency spectrum. In addition, we estimated the mean and mean squared error of F_ST_ for variants with a minor allele frequency (MAF) ≥0.05 from eight European populations (N = 158) of the Human Genome Diversity Project (HGDP) data set [31], which was also used in our RMSE calculations. Note, one of each related pair in the HGDP samples was removed, and imputation of missing genotypes was performed as previously described [32].

To calculate these statistics in the exome data, we divided the genome into 1 Mb windows. Ten of these windows were randomly selected, with replacement, and concatenated to form a “genomic region”. This procedure was repeated 10,000 times in order to estimate a genome-wide distribution for the various statistics to compare to the simulated data. In the simulated data, we followed a similar procedure by calculating the statistics defined above on ten 1 Mb windows. In total, we performed 21 independent replicates in order to get a good estimate of the parameters and allow for variation in the number of segregating sites per region (see below). The average value of each statistic was used to calculate the RMSE. The RSME function was calculated similarly to that described by Schaffner et al. [30]. Specifically, for RMSE the *i* ^th^ statistic is:
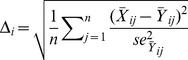
(4)where 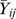 is the mean of the 10,000 replicates and 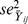 is the mean squared error. Some of the statistics are distributions of values, and we used different number of bins to summarize the distributions (r^2^ by physical genomic distance [*n* = 10] and the site frequency spectrum by minor allele frequency [*n* = 7]), which were indexed by *j* in Equation 4. For the other statistics, *n* = 1. 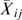 is the average for the simulated data of the 21 replicates for statistic *i* bin *j*. The combined RMSE across all statistics is:



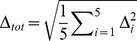
(5)Optimization was performed using a genetic algorithm until no further improvement in the RMSE was observed for 10 iterations.

We also performed Kolmogoroff-Smirnov (K-S) tests to compare the distributions of the number of segregating sites in the observed and simulated data. To achieve a better fit for this statistic, the scaled population mutation rate *θ = 4N_e_µ* was defined by two parameters, an average (*µ*) and a deviation from that average (*δ*) using the function *µk = µ+(((np –1)/2)-k)*×*δ* where window *k* had a mutation rate of *µ_k_* and *n_p_* are the number of replicates. We used an odd number (i.e. 21) so that *µ*_k_ = *µ* for one replicate and the other values were centered on *µ.*

### Coalescent Simulations To Evaluate the SAR and Power

Using the parameter values inferred as described above (see Figure 1B), we simulated 10,000 haplotypes (2,000 from each of the five subpopulations) using coalescent simulations with MSMS [33] of length 41 kb, the average gene size in genomic coordinates. This resulted in 1,000 diploid individuals from each subpopulation and 5,000 individuals overall.

We selected 1,000 cases and 1,000 controls using Equation 3, which requires specifying the disease risk of individuals in the *i* ^th^ subpopulation, *P(d = c|i),* and the proportion of individuals sampled from each subpopulation, *P(i)*. We varied these parameters over a wide range of values. Specifically, we set *P(d = c | i = 1) = 0.03+Y*, *P(d = c | i = 2) = 0.03+Y/2*, *P(d = c | i = 3) = 0.03*, *P(d = c | i = 4) = 0.03-Y/2*, and *P(d = c | i = 5) = 0.03-Y*, and considered values of *Y* = [-0.03, -0.02, …,0.03]. When *Y* = 0 all subpopulations have a disease prevalence of 0.03. Similarly, to define the proportion of individuals from each subpopulation we set *P(i = 1) = 0.2+X, P(i = 2 = 0.2+X/2*, *P(i = 3) = 0.2*, *P(i = 4) = 0.2-X/2*, and *P(i = 5) = 0.2-X* and considered values of *X* = [−0.2, −0.1, …, 0.2]. When *X* = 0, individuals in all subpopulations have a 20% chance of being sampled. All pairwise testing of *X* and *Y* values results in 35 distinct combinations. The condition of *Y* = 0 and *X* = 0 is equivalent to the situation of “no confounding” with population structure [2]. We evaluated each set of parameters with 1,000 “gene” regions and estimated the proportion significant at the α = 0.05 level.

We also evaluated the power of logistic regression based methods, which can incorporate covariates. To this end, we used the same simulations generated with the five-population model and the same population risk confounding framework (see Figure S1C in File S1), but reduced the number of parameters by fixing *P(i)* = 0.2. In order to generate case/control status, we used a logistic regression model where variants with a MAF≤1% could modify risk with odds ratios (ORs) of 1.0, 1.5, 2.0, 3.0, 4.0, or 5.0. After all individuals in each subpopulation were assigned a case/control status from the logistic regression, we selected the number of cases and controls from each subpopulation in agreement with the proportion given by Equation 3. This process was replicated for 500 regions, each with a minimum of five rare variants. We also evaluated the power and SAR with zero, one, or ten PCs with four tests (T1, CMC, StepUp, and StepDown) that could incorporate covariates. Five rare variants was used so that each segment had similar potential of being significant.

To explicitly evaluate how the magnitude of population structure influences the SAR, we also considered a simple model with two subpopulations and varied the time of population splitting (see Figure S1D in File S1). Specifically, we considered six-generation times of population splitting that span the range estimated in the calibrated model (4N_e_×[1.5, 2.0, …4.0]×10^−3^) as well as a divergence time of zero (i.e. a single panmictic population). We also included the migration rate estimate and other parameters of the calibrated model. For each time of population splitting, we simulated 4,000 haplotypes (2,000 diploid individuals) in each of the two populations. We then sampled 1,000 cases and 1,000 controls from Equation 3 by fixing the values of *P(i)* to 0.5 for each subpopulation and calculating disease risks according to the equations *P(d = c|i = 1) = 0.02+X* and *P(d = c|i = 2) = 0.02-X* for values of *X* = [0.000, 0.005, …, 0.020].

### Analysis of Spurious Associations in a Large Exome Dataset

To empirically evaluate the SAR, we analyzed exome sequences from a sample of 4,298 European Americans with modest, but statistically significant, levels of population stratification (http://snp.gs.washington.edu/EVS/) [17]. We sampled 1,000 cases and 1,000 controls from the dataset such that the probability of being a case is a function of where the individual is located in PC space. Thus, individuals that cluster together in PC space have similar likelihoods of being cases (or controls), thus mimicking the effect of population structure. To this end, we used a logistic regression approach to generate phenotype affection status probabilities based on the first two PCs (Equation 6) and determined case/control status by comparing the probability to a uniformly distributed random number:
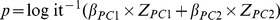
(6)


To calculate the *β* values, which are measured in unit changes of PC values (i.e., *Z_PC1_* and *Z_PC2_*) we used the equation:
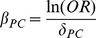
(7)where δ_PC_ is a function of the distance between the minimum and maximum values along a PC axis and allows us to vary the strength of PC confounding, and OR denotes the odds ratio. Values of δ_PC_ considered were 1, ½, or ¼ of the intervening distance between the most distant individuals, and smaller values indicate larger differences in disease risk among individuals in PC space.

For each combination of δ_PC_, OR, and PC, we performed ten replicates of an exome-wide analysis with the logistic T1 calculated for each gene. In total, we performed 490 exome scans with a median gene count of 14,360 (min = 14,313 and max = 14,401) where differences in gene number are due to the individuals sampled and a minimum of five rare variants per gene.

## Results

### A Calibrated Demographic Model

Through extensive simulations, we inferred parameters of a demographic model that recapitulate patterns of variation present in observed exome data (Figure S1A in File S1). The calibrated demographic model has an average RMSE of 1.42, which is similar to the values produced by Schaffner et al. [30]. The average p-values for the number of segregating sites from the K-S test was 0.94, consistent with a good fit between the simulated and empirical distributions. The parameters estimated from this procedure were then compared to an independent set of 316 European American individuals sampled from ESP, which verified the consistency of the parameter estimates (mean RMSE = 2.5148; average K-S p-value = 0.72). As expected these values were slightly different from the original data set, but are still within acceptable limits. All subsequent simulated data were generated from parameter values of our calibrated demographic model (Table S2 in File S1).

### Fine-scale Population Structure Leads to Spurious Associations

Using the calibrated demographic model (Figure S1B in File S1), we simulated 1,000 cases and 1,000 controls, and evaluated the SAR of nine rare variant association methods for data simulated under the null hypothesis of no causal disease variants. As described in Materials and Methods, the level of confounding is determined by differences in disease prevalence among subpopulations (which varied from 0% to 6% as determined by the variable *Y*) and the proportion of individuals sampled from each subpopulation (which varied from 0% to 40% as determined by the variable *X*). For example, when Y = –0.03, the disease prevalence is 0, 0.015, 0.03, 0.045, and 0.06 in subpopulations one through five, respectively. Similarly, when X = -0.2, the proportion of individuals sampled from subpopulations one through five are 0, 0.10, 0.20, 0.30, and 0.40, respectively.

**Figure 2:**
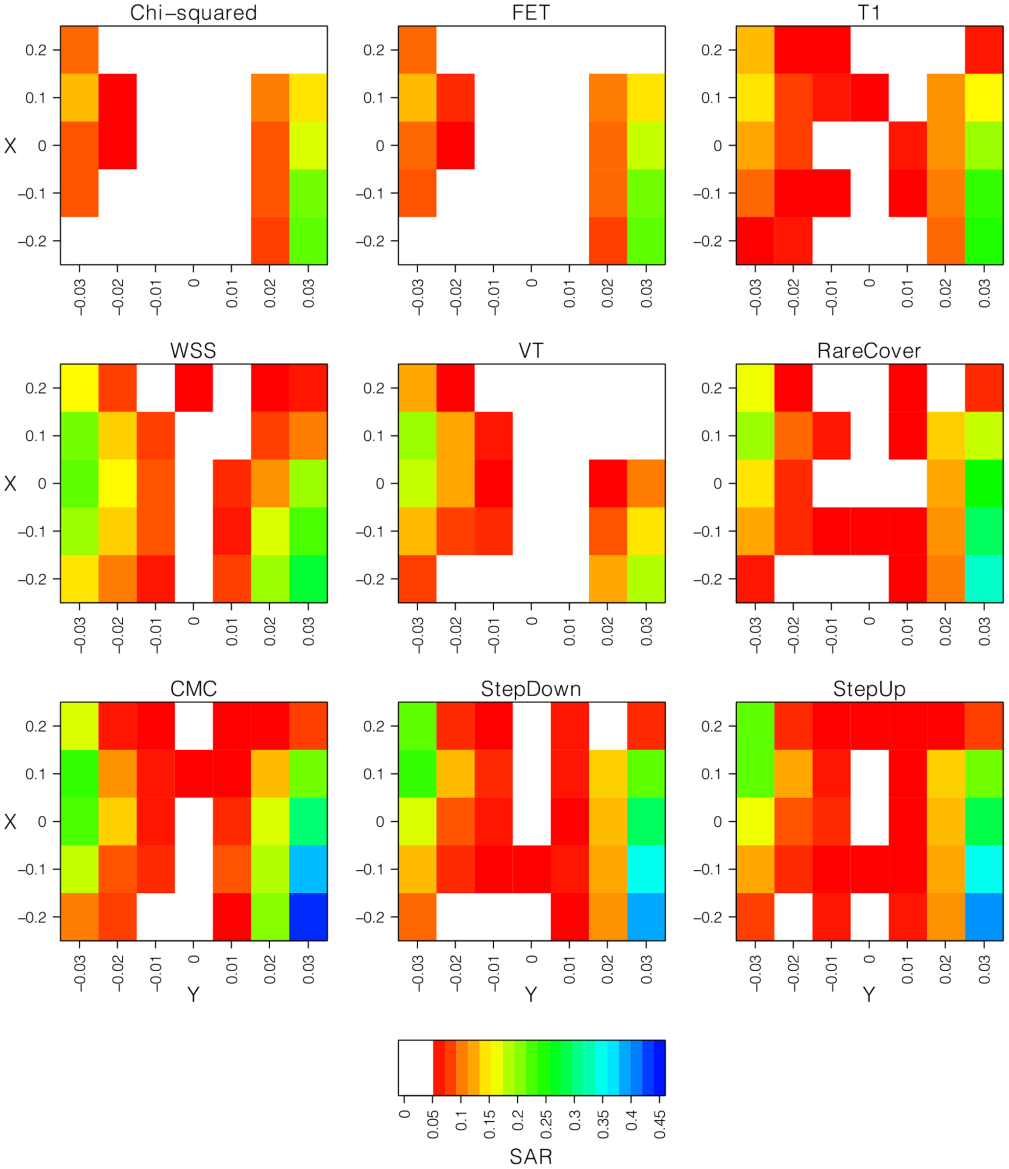
Rare variant association methods exhibit higher than expected rates of spurious associations. Each square represents a confounding scenario set by different values of disease risks, parameterized by *Y*, and the proportions of each sampled subpopulation, parameterized by *X* as presented in the text. A value of 0.0 for *X* indicates an equal proportion of each subpopulation in the study pool and 0.00 for *Y* indicates an equal disease risk. Spurious association rates (SAR) lower than 5% are represented as white, with other levels signified by sequential coloration with red the lowest and blue the highest. Actual values of the SAR can be found in Figure S2 in File S1.

Simulations where the disease prevalence was identical and individuals were sampled equally from all subpopulations (X = 0 and Y = 0) yielded expected type I error rates (Figure 2; see also Figure S2 in File S1). However, even relatively small differences in disease prevalence in the presence of fine-scale population structure can lead to elevated rates of spurious associations. For example, each of the nine methods had an elevated SAR with differences in disease prevalence as low as 4% among subpopulations (*Y*≤–0.01 and Y≥0.01; Figure 2). The logistic CMC, which simultaneously tests common and rare variation, had the highest levels of spurious associations (a maximum SAR of 43.4% when *Y* = 0.03 and *X* = −0.2; Figure 2). Although common variants may contribute to the observed spurious associations, some of the optimization based association methods that only examine rare variants have SARs comparable to CMC (maximum SARs of 39.3% for StepUp and 32.4% for RareCover). Notably, these optimization methods also have the lowest power to detect rare variant associations (see Figure S3 in File S1).

**Figure 3:**
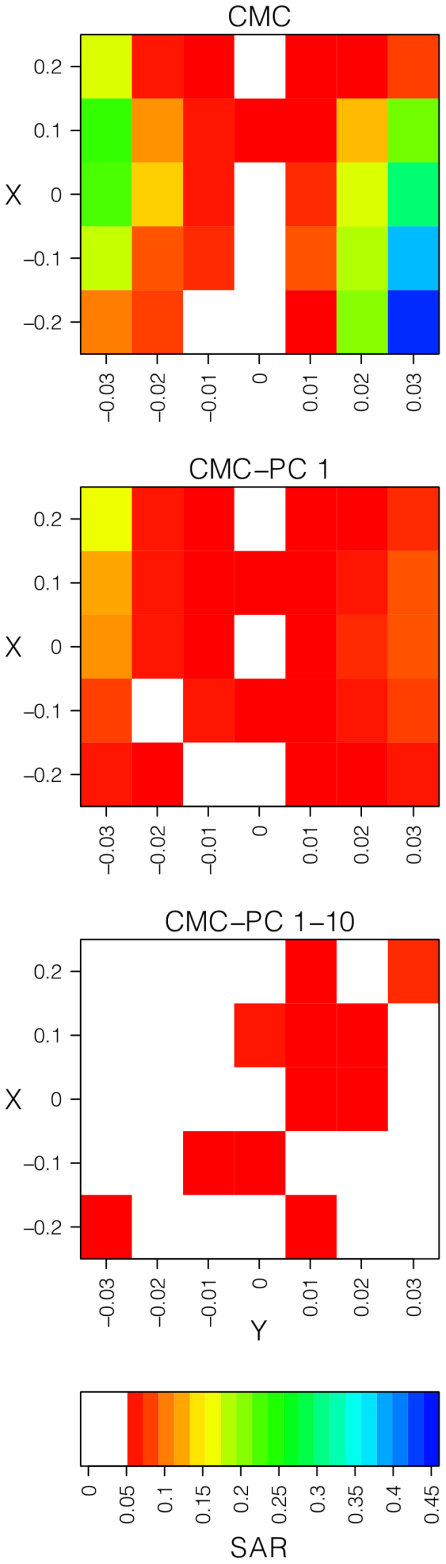
The effects of PCA correction on logistic CMC. The top figure has the spurious association rate (SAR) of CMC without correcting for population structure. The middle figure shows the SAR of CMC when a single PC is included as a covariate. The bottom figure shows the SAR of CMC when 10 PCs are included as covariates. Each square represents a confounding scenario parameterized by *X* and *Y* as presented in the text. SAR lower than 5% are represented as white, with other levels signified by sequential coloration with red the lowest and blue the highest. Actual values of the SAR can be found in Figure S4 in File S1.

Among the association methods considered here, the logistic regression based methods (i.e. T1, CMC, StepUp, and StepDown) are able to incorporate covariates. By including ten PCs, the SAR for each of these four methods was reduced to nominal levels. As an example, Figure 3 shows the PCA corrected results for CMC with zero, one, or ten PC covariates (see also Figure S4 in File S1). Results from the other three methods are similar and presented in Figure S5 and S6 in File S1. Additional numbers of PCs were explored, but were not necessary to correct for population structure in our demographic model (data not shown). We note that selecting the optimal number of PCs to include as covariates is a difficult problem and the strategy to correct for structure depends on the demographic scenario and disease risk differences between groups [34].

We also evaluated the T1 test at a lower p-value threshold (α = 0.0001) and found comparable results (see Figure S7 and S8 in File S1). Again, the correction with 10 PCs brought the SAR to within the expected range. The 95% confidence interval included 0 of 1000 replicates for all parameter values tested.

### Correcting for Spurious Associations Reduces Power of Rare Variant Association Methods

**Figure 4:**
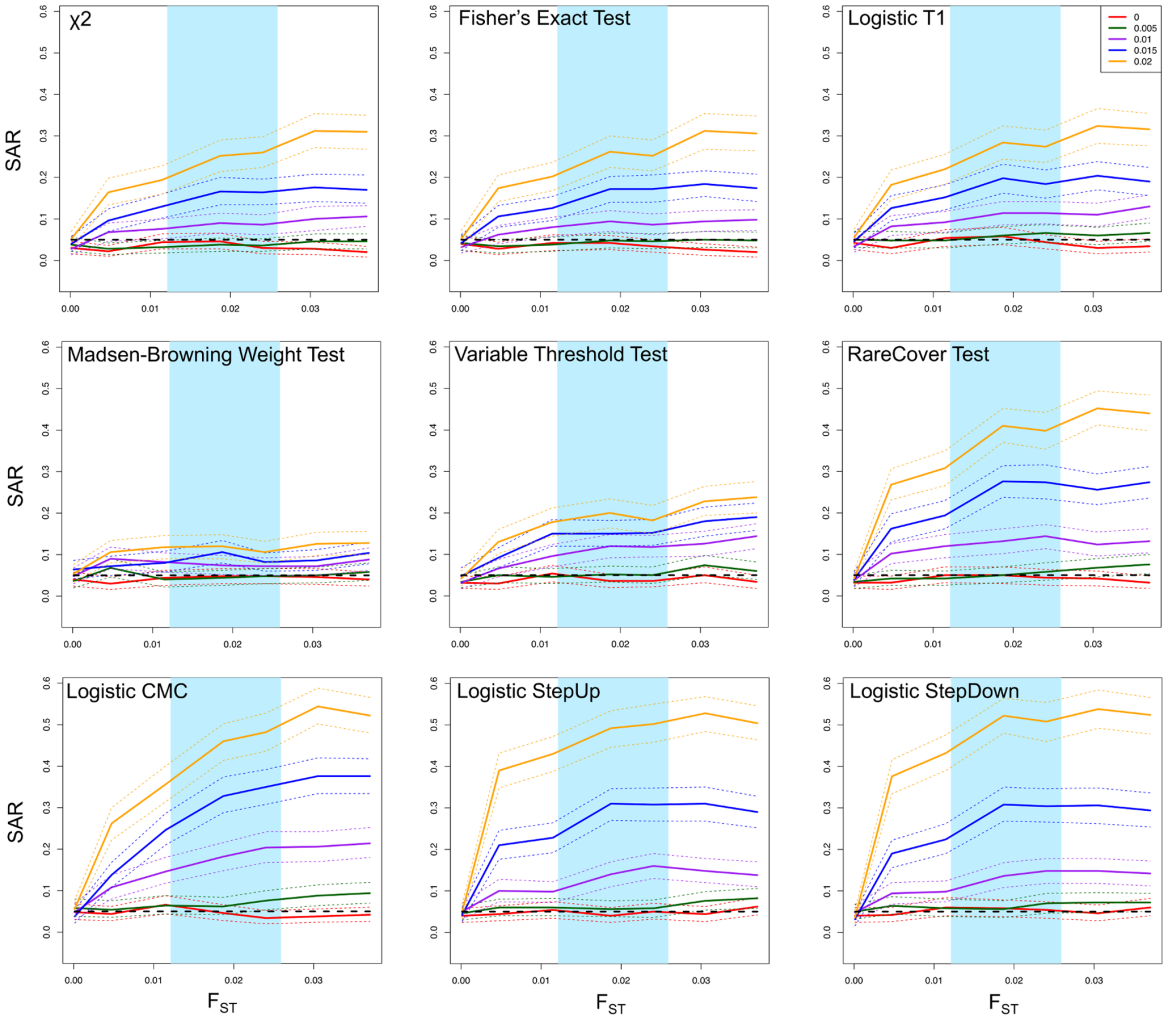
SAR of rare variant association methods as a function of F_ST_ . We tested for spurious association rates at various divergence times, presented as F_ST_ estimates for comparison with European populations in HGDP (light blue shading). The various lines represent differences in disease risk according to the equations *P(d = c|i = 1) = 0.02+ X* and *P(d = c|i = 2) = 0.02 − X*. The dashed black line represents the α = 0.05 value used to determine significance and the dotted lines represent the 95% confidence intervals calculated by bootstrapping.

We next tested how correcting for population structure influences power of rare variant association methods (Figure S1C in File S1). We focused this analysis on the four logistic regression based methods as they can incorporate PC covariates. All four methods had higher power in simulations without population structure (and no PC correction), and in some cases significantly so (Figure 4). Intuitively, this makes sense as correcting for confounding can mask true signal in cases where causal variants and confounding are correlated. For example, all methods incurred the greatest loss of power when levels of confounding were higher (Figure 4).

Nonetheless, it is still possible to have good power in samples with population structure. As expected, the logistic T1 test performed the best, as this is the same model we used to generate the genotype-phenotype map. CMC also performed well, but the StepUp and StepDown optimization methods had the lowest power compared to the other methods, consistent with our estimates of power in the absence of population structure (Figure S3 in File S1).

### Levels of Population Structure Necessary to Elevate the SAR

**Figure 5:**
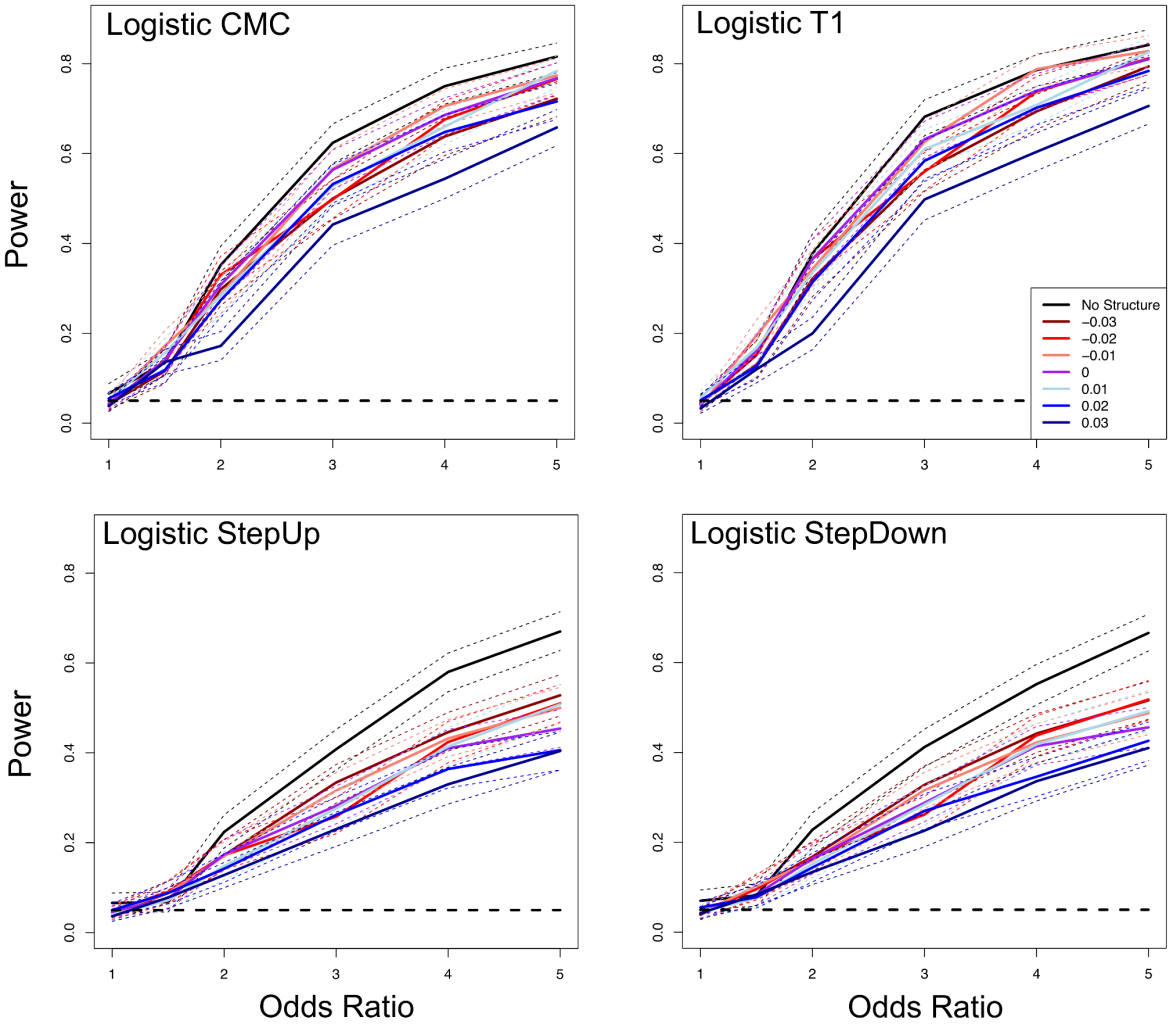
Correcting for population structure reduces the power of rare variant association methods. The figure shows the power of logistic regression methods when including ten PC covariates. The x-axis shows the odds ratio (OR), where 1.0 is the null model. “No Structure” indicates simulations where power was estimated from sampling cases and controls from a single panmictic population, but still corrected for structure. The dashed black line represents α = 0.05 and the dotted lines represent the 95% bootstrap confidence intervals.

To more precisely delineate the magnitude of population structure necessary to inflate the SAR, we considered a simpler demographic model of two subpopulations and varied the time of population splitting (see Materials and Methods; Figure S1D in File S1). All nine rare variant association methods exhibited the expected type I error rate in the absence of population structure or when the difference in disease prevalence was less than 1% among subpopulations (Figure 5). However, with larger differences in disease prevalence, the SAR can be substantially inflated, even for levels of divergence less than or equal to that observed in European populations within the HGDP (F_ST_ in the range of 0.01–0.025; light blue shading in Figure 5). For example, with a difference in disease prevalence of 3%, very low levels of differentiation (F_ST_ ∼ 0.005), RareCover, StepUp, StepDown, CMC, and the WSS have SARs of 0.16, 0.21, 0.19, 0.14, and 0.07, respectively. Thus, these results help refine the conditions in which spurious associations become an important issue to rare variant association analyses. For the logistic regression models, we evaluated the SAR when one or ten PCs were included as covariates. These methods recovered reasonable error rates with a single PC (Figure S9 in File S1).

### SAR in a Large European American Exome Dataset

The NHLBI Exome Sequencing Project recently described a large, high-quality sequence data set consisting of exomes (approximately 15,000 protein-coding genes) from 4,298 European Americans and 2,017 African Americans [17]. As the median coverage of this dataset was over 100x, even very rare genotypes were called accurately. To complement the simulations described above, we empirically assessed the SAR in the European American samples using the logistic T1 method. We focused on this method because it is a widely used statistic that is similar to several other approaches such as CMC [15], [16], [20] and is computationally efficient.

**Figure 6:**
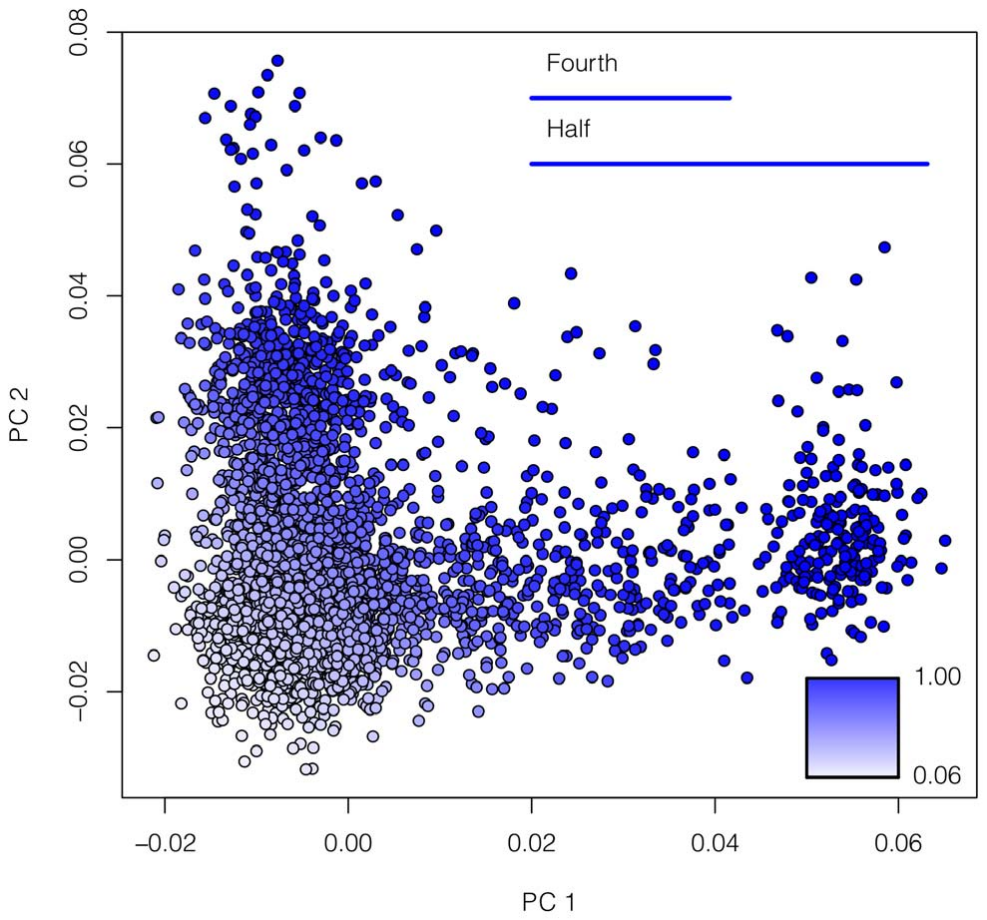
Probability of being a case as a function of PC1 and PC2. Individuals (dots) are colored according to the logistic regression with β values scaled so that for this example an odds ratio (OR) of 5 for a distance of a fourth of the minimal and maximal values for each axis. In other words, individuals separated by a fourth of the PC distance will have an OR of 5 compared to each other. The probability of being a case is thus indicated by the color of each dot on a scale from 0.06 to 1, as indicated by the gradient (lower right corner).

We generated phenotypes that are confounded with population structure using a PCA approach as described in Materials and Methods. For example, Figure 6 shows the probability of being a case for each of the 4,298 European American individuals, assuming that individuals separated by a fourth of the maximal PC1 or PC2 distance have an OR = 5 of being a case. After assigning phenotypes, we randomly selected 1,000 cases and 1,000 controls from the European American individuals and calculated the logistic T1 statistic on each gene that contained a minimum of five rare variants. We repeated this analysis ten times for each of the parameter settings as described in Materials and Methods.

The highest average SAR value from these scans was 7.07%, which is only slightly elevated above the expected value of 5%. We did not attempt to correct this SAR using PCA as that was how the confounding was generated. Even with the most extreme parameters considered in Table 1, the SAR of this European American sample is unlikely to be problematic for the sample size considered here and is likely due to the limited genetic differentiation among individuals. For example, the F_ST_ between extreme groups from the first and second PC (as identified in Figure S10 in File S1), which have a maximal average F_ST_ of 0.011, is lower than the minimum pairwise F_ST_ observed from the HGDP populations of 0.012. However, we note that our simulations suggest that with larger sample sizes, and hence higher power to detect structure, the magnitude of population structure present in European Americans could result in elevated rates of spurious associations.

**Table 1:** Spurious association rates in the exome data.

	PC1						
PC2	OR 1/5 Fourth	OR 1/5 Half	OR 1/5 Full	OR 1	OR 5 Full	OR 5 Half	OR 5 Fourth
OR 1/5 Fourth	0.0686	0.0548	0.0448	0.0400	0.0421	0.0501	0.0660
OR 1/5 Half	0.0683	0.0543	0.0442	0.0402	0.0425	0.0521	0.0680
OR 1/5 Full	0.0686	0.0544	0.0441	0.0397	0.0443	0.0524	0.0684
OR 1	0.0700	0.0555	0.0442	0.0389	0.0434	0.0527	0.0707
OR 5 Full	0.687	0.0522	0.0434	0.0391	0.0454	0.0541	0.0684
OR 5 Half	0.0683	0.0530	0.0441	0.0395	0.0444	0.0530	0.0689
OR 5 Fourth	0.0641	0.0494	0.0401	0.0393	0.0448	0.0539	0.0667

The values are the average spurious association rate for ten run using 1,000 cases and controls from the European Americans in the Exome Sequencing Project. These are the rates at the 5% significance threshold for parameters defined as odds ratios (ORs) of 1/5, 1, or 5 for a fourth, half, or full distance between the minimum and maximum for each axis: PC1 are the columns, and PC2 are the probabilities calculated for each individual. Smaller values indicate larger differences in disease risk among individuals in PC space.

## Discussion

We have demonstrated that all rare variant association methods considered here can yield elevated rates of spurious associations in the presence of fine-scale population structure. Furthermore, we showed that incorporating PCs as covariates can mitigate the confounding effects of population structure and return spurious association rates to be within normal type I error rates. The ability of PCA to correct for spurious associations in our demographic model is possibly attributable to the fact that rare and common variants possess correlated patterns of population structure (unpublished data). In demographic models where this is not true, PCA may not be sufficient to properly control for spurious associations [20]. An alternative strategy for attenuating the effects of population structure in rare variant association methods is to carefully match population proportions in cases and controls, and disease risks in subpopulations [2], [35], [36]. This occurred in our simulations when *X* = *Y* = 0, and did not have elevated SARs. However, matching may not always be feasible, and is particularly difficult in situations where subtle differences in structure and disease prevalence exist among unidentified subpopulations. Although levels of confounding in these scenarios are weak, the very large sample sizes necessary to robustly detect associations with rare variants create the conditions necessary to generate spurious associations.

The differences in disease risk among populations that we found to generate increased SARs are plausible, and further underscore the importance of carefully designing and interpreting rare variant association methods. For instance, between populations of European men there is a 2.5% to >10% difference in rates of lung cancer, though a less striking difference among women [37]. Many other examples exist, such as differences in prevalence of diabetes (ranging from 1.6% to 3.1% [38]) and Cystic Fibrosis (ranging from 0.001% to 0.03% [39]). Note, it is not necessary that differences in disease risk be genetic, only that they exist and are confounded with population structure. For example, the true cause of differing levels of lung cancer risk could be something other than a genetic predisposition (e.g. differences is acceptance of smoking between cultures), but would still be confounded with population genetic structure.

Another issue for rare variant association methods is admixture. Discrete populations, as we have modeled here, can be viewed as a special case of an admixture model [29] where an admixed individual’s probability of carrying an allele and having a phenotype would be a weighted average of their source populations’ values. In contrast to common variation, rare variation is more likely to be population specific [17], [22] and subject to confounding. In addition, because admixture proportions vary widely both among individuals and within an admixed genome, global corrections such as PCA are unlikely to fully address the heterogeneity in the strength of confounding across loci. Clearly, additional studies are needed to better delimit the effects of admixture on rare variant association methods, and optimal methods for mitigating confounding.

In conclusion, although rare variant association tests are poised to provide new insights into the genetic architecture of complex traits, they are susceptible to spurious associations when individuals are sampled from even modestly differentiated populations. All methods considered here showed elevated SARs, suggesting this is a general phenomenon that should be considered in the design, analysis, and interpretation of rare variant association studies.

## Supporting Information

File S1 **This file contains additional figures and tables that support the conclusions made in the main text.** It also contains a detailed explanation of the ESP Banner and an extended acknowledgements section.
(PDF)
